# ADAMTS5 in Osteoarthritis: Biological Functions, Regulatory Network, and Potential Targeting Therapies

**DOI:** 10.3389/fmolb.2021.703110

**Published:** 2021-08-09

**Authors:** Lejian Jiang, Jiachen Lin, Sen Zhao, Jiaqian Wu, Yongming Jin, Li Yu, Nan Wu, Zhihong Wu, Yue Wang, Mao Lin

**Affiliations:** ^1^ Department of Orthopedic Surgery, The First Affiliated Hospital, Zhejiang University School of Medicine, Hangzhou, China; ^2^ Spine Lab, The First Affiliated Hospital, Zhejiang University School of Medicine, Hangzhou, China; ^3^ State Key Laboratory of Complex Severe and Rare Diseases, Department of Orthopedic Surgery, Peking Union Medical College Hospital, Peking Union Medical College and Chinese Academy of Medical Sciences, Beijing, China; ^4^ Beijing Key Laboratory for Genetic Research of Skeletal Deformity, Beijing, China; ^5^ Medical Research Center, Peking Union Medical College Hospital, Peking Union Medical College and Chinese Academy of Medical Sciences, Beijing, China; ^6^ Department of Operating Room, The First Affiliated Hospital, Zhejiang University School of Medicine, Hangzhou, China; ^7^ Key Laboratory of Big Data for Spinal Deformities, Chinese Academy of Medical Sciences, Beijing, China

**Keywords:** ADAMTS5, osteoarthritis, signaling pathways, monoclonal antibody, small molecule inhibitors, RNA therapies

## Abstract

ADAMTS5 is involved in the pathogenesis of OA. As the major aggrecanase-degrading articular cartilage matrix, ADAMTS5, has been regarded as a potential target for OA treatment. We here provide an updated insight on the regulation of ADAMTS5 and newly discovered therapeutic strategies for OA. Pathophysiological and molecular mechanisms underlying articular inflammation and mechanotransduction, as well as chondrocyte hypertrophy were discussed, and the role of ADAMTS5 in each biological process was reviewed, respectively. Senescence, inheritance, inflammation, and mechanical stress are involved in the overactivation of ADAMTS5, contributing to the pathogenesis of OA. Multiple molecular signaling pathways were observed to modulate ADAMTS5 expression, namely, Runx2, Fgf2, Notch, Wnt, NF-κB, YAP/TAZ, and the other inflammatory signaling pathways. Based on the fundamental understanding of ADAMTS5 in OA pathogenesis, monoclonal antibodies and small molecule inhibitors against ADAMTS5 were developed and proved to be beneficial pre-clinically both *in vitro* and *in vivo*. Recent novel RNA therapies demonstrated potentials in OA animal models. To sum up, ADAMTS5 inhibition and its signaling pathway–based modulations showed great potential in future therapeutic strategies for OA.

## Introduction

Osteoarthritis (OA) is one of the most common chronic joint lesions, mainly affecting people aged 50–75 years, with an approximate prevalence of 4–5% in the hand, 6% in the hip, and 16–17% in the knee in the general population (Hunter and Bierma-Zeinstra, 2019). OA is characterized by articular cartilage loss, subchondral bone sclerosis, and osteophyte formation (Loeser et al., 2012). Etiologically, primary OA is driven by a combination of inheritance, aging, obesity, inflammation, and biomechanical risk factors. Dysregulation of signaling pathways, especially the activation of proinflammatory pathways, promotes the overactivation of matrix-degrading enzymes and exacerbates the degradation of cartilage extracellular matrix (ECM) (Glyn-Jones et al., 2015). Collagens and aggrecan are both pivotal structural components of cartilage ECM, and their degradation is a significant event at the early stage of OA (Maldonado and Nam, 2013). It has been documented that matrix metalloproteinases (MMPs, especially MMP-13) and a disintegrin and metalloproteinase with thrombospondin motifs (ADAMTSs, especially ADAMTS4 and ADAMTS5) facilitate type II collagen and aggrecan degradation, respectively (Verma and Dalal, 2011).

ADAMTSs are a family of zinc metalloendopeptidases that participate in diverse biological processes, such as procollagen processing, ECM remodeling, inflammation, cell migration, and vascular biological processes (Kelwick et al., 2015). In particular, ADAMTS5 (aggrecanase-2) overexpression is a key risk factor in degenerative joint diseases and intervertebral disc degeneration (Wu et al., 2014; Santamaria, 2020).

ADAMTS4 and ADAMTS5 are thought to analogously mediate aggrecan cleavage. However, the protective effects of *Adamts5* gene knockout and ADAMTS5-specific antibodies in surgically induced OA mouse models emphasize that ADAMTS5 is the major aggrecan-degrading enzyme in OA (Glasson et al., 2004; Glasson et al., 2005; Apte, 2016). Therefore, ADAMTS5 has long been regarded as a potential target for OA treatment. However, as the balance between matrix synthesis and degradation is critical for ECM structure and tissue homeostasis, direct inhibition of ADAMTS5 has aroused great concern. For instance, ADAMTS5 knockout can lead to deleterious accumulation of proteoglycan in the adult cardiovascular system and disrupt aortic wall mechanics in mice (Dupuis et al., 2011; Dupuis et al., 2013; Fava et al., 2018; Dupuis et al., 2019). Recently, updated knowledge regarding ADAMTS5 regulatory factors and the preclinical discovery of potential disease-modifying drugs have provided more options for OA treatment. Thus, comprehensive insight into the biological functions and molecular regulation of ADAMTS5, supplemented by the current developmental stages of diverse classes of drugs, may be necessary to better understand the involvement of ADAMTS5 in OA and identify future therapeutic strategies.

### Functions and Regulation of ADAMTS5 in Normal Cartilage Extracellular Matrix

Aggrecan is a major component of cartilage and protects collagens against degradation (Pratta et al., 2003). Aggrecan glycosaminoglycan chains provide a gel-like structure and mechanical resistance in joints (Kiani et al., 2002). Increased levels of aggrecan fragments are a typical pathological change in cartilage and may serve as a severity indicator for OA (Roughley and Mort, 2014).

**FIGURE 1:**
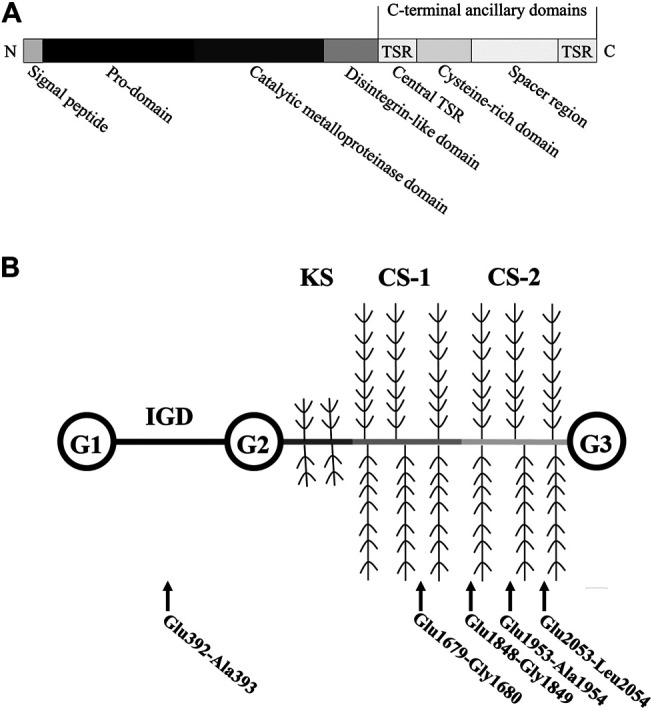
Structure of human ADAMTS5 and Aggrecan. **(A)** Human ADAMTS5 domain structure. Thrombospondin type 1 sequence repeat, central TSR. **(B)** Structure of Aggrecan. ADAMTS5-mediated cleavage within the aggrecan occurs at (glutamate) Glu-Xaa (where Xaa = alanine, glycine, and leucine) recognition motifs. Abbreviations: globular domains, G1-3; interglobular domain, IGD; keratan sulfate attachment domain, KS; chondroitin sulfate attachment domains, CS-1 and -2.

Aggrecan can be cleaved by ADAMTS family members, including ADAMTS1, ADAMTS4, ADAMTS5, ADAMTS8, and ADAMTS15 (Santamaria, 2020). Among these ADAMTSs with aggrecan-degrading activity, ADAMTS4 and ADAMTS5 tend to be most efficient (Tortorella and Malfait, 2008). These two aggrecanases are regarded as critical factors in metabolism, homeostasis, and pathological changes of joint ECM. Structurally, from the N- to C-terminus, ADAMTS5 is composed of a signal peptide, a pro-domain, a catalytic metalloproteinase domain, a disintegrin-like domain, and other C-terminal ancillary domains (a central thrombospondin type 1 sequence repeat (central TSR) motif, a cysteine-rich domain, a spacer region, and an additional TSR motif) (Kelwick et al., 2015) (Figure 1A). Extracellular excision of the pro-domain by proprotein convertases, specifically furin and furin-like enzymes, is essential for ADAMTS5 activation (Longpré et al., 2009). The catalytic metalloproteinase domain alone has little proteolytic activity, and the combination of its C-terminal ancillary domains increased its proteolytic activity (Gendron et al., 2007). The cysteine-rich domain is critical for the localization of ADAMTS5 and its binding to the cell surface and ECM (Gendron et al., 2007). Similar to ADAMTS4, the cysteine-rich domain of ADAMTS5 is also critical for its interaction with the glycosaminoglycan chains of aggrecan, and the central TSR motif is necessary for aggrecan recognition and cleavage (Tortorella et al., 2000; Flannery et al., 2002; Fushimi et al., 2008). Exosites in the spacer region are responsible for substrate recognition and proteolysis (Santamaria et al., 2019).

**FIGURE 2:**
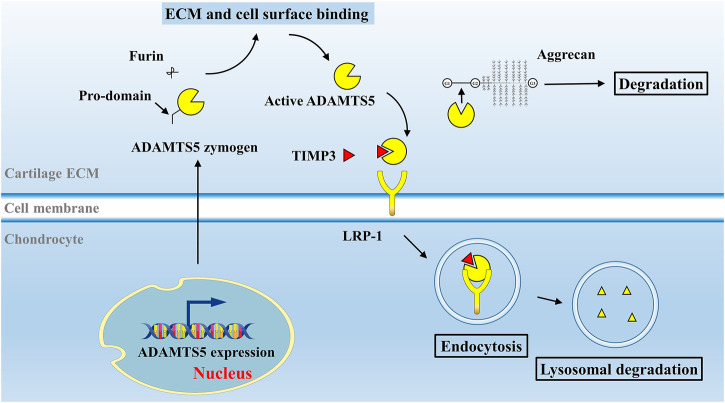
Activation and degradation mechanism of ADAMTS5 *in vivo*. In normal cartilage, ADAMTS5 is activated under the stimulation of inflammatory factors or breakdown products in cartilage ECM. After the removal of its pro-domain by furins, activated ADAMTS5 cleaves the aggrecan core protein at its specific Glu-Xaa recognition motifs. ADAMTS5 can be inhibited by its endogenous inhibitor, TIMP3. ADAMTS5, together with TIMP3 are subsequently endocytosed by chondrocyte via LRP-1 receptor and degraded. Abbreviations: tissue inhibitor of metalloproteinase 3, TIMP3; lipoprotein receptor–related protein 1, LRP-1.

ADAMTS5 is expressed at low levels in various tissues, including the placenta, heart, lung, skeletal muscle, tendon, cartilage, and synovium (Fagerberg et al., 2014). Breakdown products of cartilage ECM can enhance MMP-13 and ADAMTS5 expression and activation (Jung et al., 2019). ADAMTS5 zymogen in the ECM is inactive and can be activated extracellularly by removal of its pro-domain (Longpré et al., 2009). Activated ADAMTS5 cleaves the aggrecan core protein at its specific recognition motifs, for example, the glutamate (Glu) 373-alanine (Ala) 374 bond (Glu392-Ala393 bond in the modern nomenclature, UniPort ID P16112) in its interglobular domain, as well as other specific sites, leading to loss of integrity of aggrecan molecules (Kiani et al., 2002; Little et al., 2007) (Figure 1B). Under physiological conditions, the aggrecanase activity of ADAMTS5 in cartilage can be inhibited by its endogenous inhibitor, tissue inhibitor of metalloproteinase 3 (TIMP3) (Figure 2). TIMPs are expressed in connective tissues and play an important role in the inhibition of MMPs (Brew et al., 2000). TIMP3, with its distinct N-terminal inhibitory domain, has a strong inhibitory effect on ADAMTS4 and ADAMTS5 (Kashiwagi et al., 2001). After forming a complex with TIMP3, ADAMTS5 can therefore be cleared by chondrocytes through lipoprotein receptor-related protein 1 (LRP-1)–mediated endocytosis in cartilage tissue (Yamamoto et al., 2013) (Figure 2). In cartilage, the cysteine-rich domain and the spacer region of ADAMTS5 are involved in effective binding to the sulfated proteoglycans at the cell surface or the ECM (Gendron et al., 2007). The central TSR motif and the spacer region can also be identified by LRP-1, leading to ADAMTS5 clearance. Thus, ADAMTS5 and TIMP3 can be endocytosed independently or as a complex. LRP-1 is an important regulator of normal cartilage homeostasis, and the location and activity of ADAMTS5 are determined by competition between the ECM and LRP-1.

### ADAMTS5 in the pathogenesis of OA *In Vitro* and *In Vivo*.

Cytological studies and animal models recapitulating OA enhance the understanding of disease progress and the evaluation of therapeutic modalities. Desirable biomarkers of OA can effectively assist indications for OA stages and monitor treatment responses (Gu et al., 2019). Since aggrecan destruction in synovial fluid is a hallmark at the early stage of OA, the major aggrecanase, ADAMTS5, is identified as a potential biomarker for the prediction of OA progression (Saberi Hosnijeh et al., 2019).

Genetic polymorphisms in *ADAMTS5* in different populations were also identified to be associated with susceptibility to OA. Bioinformatic analysis on 2,715 patients with OA and 1,185 controls in a European Caucasian population identified two single-nucleotide polymorphisms at *ADAMTS5* gene loci (Rodriguez-Lopez et al., 2008). These two nonsynonymous variants appeared clustered in patients with severe OA and resulted in an aberrant amino acid sequence of encoded ADAMTS5. Furthermore, another genetic variant in *ADAMTS5*, rs2830585, was identified in a Chinese population with 300 pairs of OA patients and control subjects (Zhou et al., 2019).

ADAMTS5, as one of the key downstream responders, was upregulated in OA models *in vitro* and *in vivo* (Song et al., 2007; Johnson et al., 2016). Moreover, cartilage destruction was rescued in *Adamts5* knockout mice with posttraumatic OA, while mice with deletion of *Adamts4* developed OA (Glasson et al., 2004; Glasson et al., 2005). In the joints and serum of rats with surgery-induced OA, the expression of ADAMTS5 was markedly increased along with OA progression (Elsadek et al., 2019).

These studies suggested a critical role of ADAMTS5 in OA development and implied that ADAMTS5 can serve as not only a predictive biomarker of OA staging and prognosis but also a potential target for OA therapy. Thus, a comprehensive understanding of ADAMTS5 regulatory pathways is required.

### Signaling Pathways Regulating ADAMTS5 Expression in the OA Pathological Process

Several signaling pathways are involved in ADAMTS5 modulation in the pathophysiological process of OA, such as Runx2 signaling, Fgf2 signaling, Notch signaling, Wnt signaling, YAP/TAZ signaling, and inflammatory signaling pathways (Chia et al., 2009; Hosaka et al., 2013; Ji et al., 2016a; Deng et al., 2018; Catheline et al., 2019; Wang et al., 2019). The regulation of ADAMTS5 and the crosstalk of each signaling pathway are discussed below.

### Runx2 Signaling and Fgf2 Signaling

Runt-related transcription factor 2 (RUNX2) is a key transcription factor in osteoblast proliferation and differentiation (San Martin et al., 2009). RUNX2 is strictly expressed in the nucleus of osteoblasts and regulates the cell cycle via its oscillating level of expression (San Martin et al., 2009). Moreover, RUNX2 can respond to mechanical signals and affect bone homeostasis (Kanno et al., 2007). In human OA cartilage, high expression of RUNX2 was detected (Zhong et al., 2016; Chen et al., 2020). RUNX2 is also responsible for hypertrophic differentiation of chondrocytes, which is a characteristic change in the development of OA (Dreier, 2010; Catheline et al., 2019).

**FIGURE 3:**
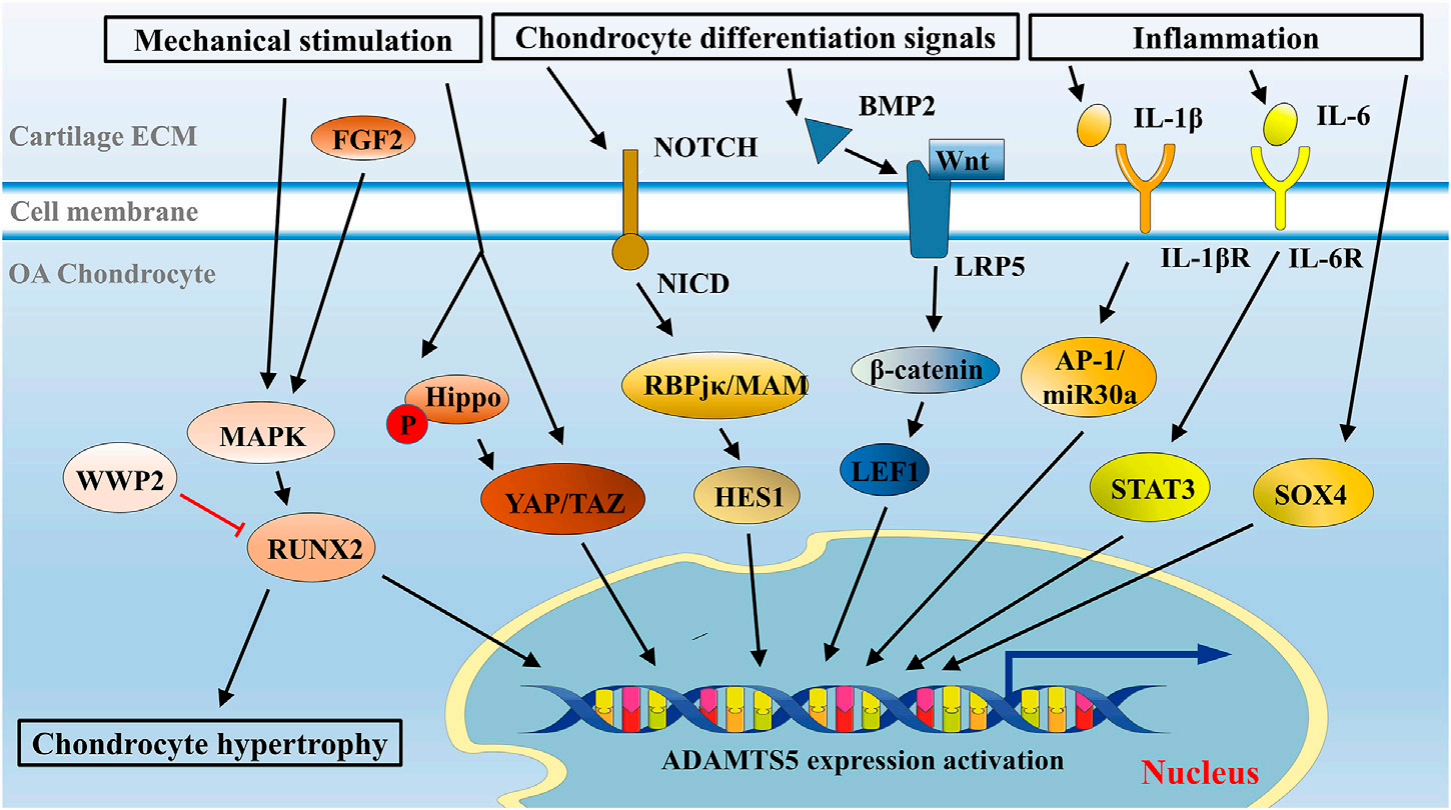
Overview of signaling network in ADAMTS5 regulation in chondrocytes. Mechanical stimulation, cell differentiation signals, and inflammatory environment are primary initiators to ADAMTS5 overexpression in OA. Signaling pathways were illustrated with recent insights, such as Runx2 signaling, Notch signaling, Wnt/β-catenin signaling, and cytokine-mediated signaling pathways, and some newly discussed signaling pathways, such as YAP/TAZ signaling and Sox4 signaling are presented in this schematic presentation.

Analysis of the *ADAMTS5* promoter sequence identified four binding sites for the RUNX family, among which RUNX2 exhibited strong affinity (Thirunavukkarasu et al., 2007). In mechanical stretch-exposed OA chondrocytes, the expression of ADAMTS5 was overactivated by RUNX2 (Tetsunaga et al., 2011). In this literature, RUNX2 might have a role as a key downstream mediator of MAPK and p38 to regulate mechanical stress–induced ADAMTS5 expression (Figure 3). This trend was also confirmed in surgically induced OA mice: the progression of OA was significantly decelerated in *Runx2* knockout mice compared with control mice (Liao et al., 2017). A decrease in the expression of ADAMTS5 was also confirmed by immunohistochemical analysis in this study (Liao et al., 2017). Recently, WW domain-containing protein 2 (WWP2), a kind of E3 ubiquitin ligase in osteoblasts, was shown to inhibit the expression of *ADAMTS5* through ubiquitination and degradation of RUNX2 (Mokuda et al., 2019).

Fibroblast growth factor 2 (FGF2), a growth factor involved in many biological processes, is implicated in chondrocyte differentiation and maintaining cartilage homeostasis and is highly associated with the severity of OA (Ellman et al., 2013; Yan et al., 2012). RUNX2 can be activated by FGF2 (Qi et al., 2020; Ji et al., 2016b). FGF2 molecules can elicit RUNX2 activation through the MAPK/ERK pathway and eventually modulate ADAMTS5 (Ji et al., 2016b; Xiao et al., 2002) (Figure 3). Notably, in human OA chondrocytes treated with FGF2 for a short time (mostly less than 1 h), FGF2 was shown to inhibit *ADAMTS5* expression and thus retard cartilage destruction (Sawaji et al., 2008), while after long-term treatment (more than 2 h), FGF2 was likely to activate RUNX2-mediated ADAMTS5 upregulation (Ji et al., 2016b). This effect may be responsible for the temporal expression pattern of ADAMTS5 in OA, although the detailed mechanism remains unclear.

### Yes-Associated Protein/TAZ Signaling

Yes-associated protein (YAP)/transcriptional coactivator with PDZ-binding motif (TAZ) signaling are important not only for mediating tissue growth, cell fate, and tissue morphogenesis but also in the development of cartilage (Vanyai et al., 2020). YAP signaling is regulated by upstream Hippo-dependent and independent signaling, such as mechanical cues, metabolic signals, and other signaling pathways (Dupont, 2016). YAP can inhibit chondrocyte maturation by suppression of Collagen type X alpha 1 chain (*COL10A1*) expression through interaction with RUNX2 (Deng et al., 2016).

Overexpression of YAP was observed in cultured chondrocytes and surgery-induced animal OA models (Gong et al., 2019). Furthermore, inhibition of YAP can reduce interleukin-1β (IL-1β)–induced expression of MMP13 and ADAMTS5 and retard cartilage degradation in OA mice (Gong et al., 2019). While inhibition of YAP expression can ameliorate osteoarthritic cartilage degradation, other studies have revealed that YAP plays a protective role as an inflammatory inhibitor in the progression of OA (Deng et al., 2018). In addition, both *Yap* knockout and overexpression of YAP promote cartilage disruption, indicating that YAP regulates cartilage homeostasis in a biphasic manner (Deng et al., 2018; Zhang et al., 2019; Vanyai et al., 2020).

Hippo signaling is triggered by mechanical stimulation and phosphorylates its downstream effectors YAP/TAZ. Mechanical stimulation can also mediate YAP activity in the Hippo-independent signaling pathway, which requires Rho GTPase activity and tension of the actomyosin cytoskeleton (Dupont et al., 2011). The opposite effects triggered by mechanical inputs converge on the regulation of YAP/TAZ. Unphosphorylated YAP/TAZ is transported into the nucleus to promote downstream ADAMTS5 transcription, while phosphorylated YAP/TAZ is degraded in cytoplasm (Zhao et al., 2011) (Figure 3).

### Notch Signaling

As a juxtacrine cellular signaling pathway, Notch signaling modulates cell differentiation and adult tissue homeostasis, including cartilage formation and pathology (Bray, 2016; Dowthwaite et al., 2004). In mice with surgically induced OA, Notch signaling is overactivated and participates in OA development (Saito and Tanaka, 2017). Generally, Notch signaling is initiated when the NOTCH receptor is cleaved by related proteinases after receiving signals from NOTCH ligands on adjacent cells (Kopan and Ilagan, 2009). The NOTCH receptor is a single-pass transmembrane receptor on the cell surface that is composed of an extracellular fragment, a membrane-tethered fragment and the NOTCH intracellular domain (NICD) (Chillakuri et al., 2012). Two main proteinases take part in the cleavage of the NOTCH receptor, a disintegrin and metalloproteinase 10 (ADAM10) and γ-secretase, which release NICD from NOTCH receptors. Gene transcription in the nucleus is subsequently regulated by the interaction of NICD with *trans-*acting elements, such as recombination signal binding protein for Ig kappa J (RBPjκ) and the coactivator Mastermind (MAM) (Nam et al., 2006; Wilson and Kovall, 2006) (Figure 3). Specifically, a downstream transcriptional repressor of Notch signaling, HES1, is upregulated (Kageyama et al., 2007). Once upregulated, HES1 is switched to an activator by its cofactor and directly upregulates the transcription of *Adamts5* and *Mmp13* in OA (Sugita et al., 2015) (Figure 3). In *Hes1* knockout OA mice, the expression level of ADAMTS5 and MMP13 was downregulated, and no significant histomorphometric difference was observed between OA mice and controls (Sugita et al., 2015). In addition, the joint cartilage of *Rbpjκ* knockout mice also presented OA-like histological changes, indicating a requisite role of Notch signaling in articular cartilage and joint maintenance (Hosaka et al., 2013; Mirando et al., 2013).

### Wnt Signaling

The Wnt/β-catenin signaling pathway is involved in physiological and pathological changes in articular cartilage and is also regarded as a potential therapeutic target of OA (Wang et al., 2019). Wnt comprises a diverse family of extracellularly secreted glycoproteins with various receptors. Canonical Wnt/β-catenin and noncanonical signaling pathways participate in numerous biological processes, such as cell proliferation, differentiation, cell fate determination, and tissue homeostasis (Steinhart and Angers, 2018). Accumulating evidence implies an important role for Wnt signaling in OA pathogenesis. In transgenic surgery-induced OA mice with constitutive activation of β-catenin, sustained expression of ADAMTS5 was observed (Rockel et al., 2016).

Reportedly, activation of Wnt/β-catenin signaling by bone morphogenetic protein 2 (BMP2) contributed to upregulation of ADAMTS5 and severe conditions of OA (Papathanasiou et al., 2012). The results of this study suggested that BMP2 was able to activate Wnt signaling via low-density lipoprotein receptor–related protein 5 (LRP-5), a key component involved in the canonical Wnt pathway. This Wnt pathway signaling promotes the binding of its downstream factor, lymphoid enhancer factor-1 (LEF1), to the *ADAMTS5* promoter and initiation of *ADAMTS5* transcription (Figure 3).

### Inflammatory Signaling Pathways

Cultured human chondrocytes and cartilage explants could be induced as *in vitro* OA models by inflammatory factors, such as IL-1β, tumor necrosis factor-α (TNF-α), and nuclear factor-κB (NF-κB). In those human OA models, the expression of ADAMTS5 was not significantly changed (Tortorella et al., 2001; Bau et al., 2002), suggesting constitutive expression of ADAMTS5 (Verma and Dalal, 2011; Bondeson et al., 2006; Bondeson et al., 2008). However, some studies in murine chondrocytes revealed that ADAMTS5 expression could be promoted by IL-1 (Ji et al., 2016a; Stanton et al., 2005). In human and mouse chondrocytes, ADAMTS5 might be differentially regulated. A recent study elucidated that IL-1β induced the overexpression of ADAMTS5 via the AP-1/microRNA-30a (miR-30a) axis (Ji et al., 2016a) (Figure 3). Notably, miR-30a belongs to a family of small endogenous noncoding RNAs, which play a role in posttranscriptional repression of gene expression (Miyaki and Asahara, 2012). Activator protein 1 (AP-1) elicited by IL-1β molecules can bind to the promoter of miR-30a and initiate its expression (Ji et al., 2016a). In the chondrogenic ATDC5 cell line, enhanced ADAMTS5 expression was also elicited under IL-1β treatment (Kobayashi et al., 2013).

Interleukin 6 (IL-6) is a cytokine with pleiotropic functions and is an essential initiator of inflammation and immunity (Tanaka et al., 2012). The continual overexpression of IL-6 is responsible for chronic inflammation and autoimmunity (Tanaka et al., 2014). Signal transducer and activator of transcription (STAT), mainly STAT3, is the main downstream effector element triggered by IL-6 molecules (Mihara et al., 2012). The expression level of IL-6 in serum and synovial fluid is associated with OA, and treatment of chondrocytes with IL-6 is a common method in OA model establishment (Tsuchida et al., 2012). A significant increase in ADAMTS5 expression was observed in IL-6-stimulated chondrocyte culture, as well as in mice with intra-articular injection of IL-6 (Legendre et al., 2005; Ryu et al., 2011). Recently, Latourte et al. (2017) found that IL-6 upregulated the expression of ADAMTS5 via the activation of downstream STAT3 *in vitro* (Latourte et al., 2017) (Figure 3). In addition, decreased expression levels of ADAMTS5 and decreased severity of OA were observed in both systemic inhibition of IL-6 and STAT3 blockade in a surgically induced OA mouse model (Latourte et al., 2017). This study provided strong evidence that ADAMTS5 can be upregulated via the IL-6/STAT3 pathway under the inflammatory conditions in OA.

NF-κB is a transcription factor stimulated by cytokines and ECM fragments in OA. NF-κB has long been recognized as a potential therapeutic target in OA (Rigoglou and Papavassiliou, 2013). There are three NF-κB binding motifs in the promoter of *ADAMTS5*, −1,196/−1,187 bp region, −896/−887-bp region, and −424/−415-bp region (Kobayashi et al., 2013). p65, also known as RelA, is one of the five components that form the NF-κB transcription factor family (Chen and Greene, 2004). Specific binding between p65 and NF-κB binding motifs in the *ADAMTS5* promoter suggested a transcriptionally induction of *ADAMTS5* expression during osteoarthritis development (Kobayashi et al., 2013). While NF-κB signaling is known to take part in inflammation in OA, it also responds to excessive mechanical loading and accelerates OA progression. Gremlin-1 is an inhibitor of BMPs and can be induced by mechanical stretch. Gremlin-1 activated by excessive mechanical loading can activate NF-κB signaling, resulting in the induction of ADAMTS5 (Chang et al., 2019).

### Other Involved Pathways

Sex‐determining region Y‐box 4 (SOX4) belongs to the SOXC subgroup of the SOX family and is a transcription factor involved in embryonic development and cell fate determination (Moreno, 2020). It has been reported that SOXC family members play a role in skeletal development (Lefebvre and Bhattaram, 2016). Overexpression of ADAMTS4 and ADAMTS5 can be induced by SOX4 in an inflammatory environment and mechanical stress in chondrogenic cell lines (Takahata et al., 2019). Chromatin immunoprecipitation assays showed that SOX4 molecules directly bound to the promoter sequences of *ADAMTS4* and *ADAMTS5* and modulated their transcription (Takahata et al., 2019). In skeletogenesis, SOX4 is involved in the promotion of canonical and noncanonical Wnt signaling, which is vital in OA (Bhattaram et al., 2014; Kato et al., 2015). Retinoic acid, with the ability to potentiate inflammatory cytokines, is commonly used to mimic OA in chondrocyte cell lines (Davies et al., 2009). In superficial zone cells of articular cartilage treated with retinoic acid, SOX4 expression was markedly induced (Takahata et al., 2019). However, *trans*-acting elements of *SOX4* in OA have not been identified. Further studies are required to explore the mechanisms controlling SOX4 in OA.

These signaling pathways are not mutually independent but form a complex network in ADAMTS5 regulation through their interactions. For example, inhibition of YAP significantly enhances the expression of RUNX2 in chondrocyte differentiation, while YAP overexpression significantly downregulates the expression of RUNX2 (Zhang et al., 2019). TAZ also participates in FGF2 signaling and activates RUNX2-mediated transcription of targeted genes (Byun et al., 2014). The Wnt/β-catenin pathway and Hippo/YAP signaling pathway can both be activated by Piezo1/2-mediated mechanical signals in joints (Zhou et al., 2020).

In this section, we assume that the activation of ADAMTS5 is a converged output of a complex molecular network including mechanical loading responses, chondrocyte differentiation, and inflammatory responses (Table 1).

**TABLE 1:** Signaling pathways that involve in ADAMTS5 regulation in the pathophysiological process of OA.

Signaling	Biological processes	Mechanism	Reference
**Runx2 signaling**	Mechanical stimulationHypertrophic differentiation	RUNX2 is a downstream target of p38 and MAPK, and can bind to the promoter sites of *ADAMTS5* and regulate its expression	Kanno et al. (2007)
		WWP2 can repress the expression of ADAMTS5 through ubiquitination and degradation of RUNX2 in osteoblasts	Mokuda et al. (2019)
**FGF2 signaling**	Chondrocyte differentiation	FGF2 can elicit RUNX2 activation through MAPK/ERK pathway and modulate ADAMTS5 expression	Ji et al. (2016a)
**YAP/TAZ signaling**	Mechanical stimulation	Unphosphorylated YAP/TAZ, mediated by both hippo-dependent and independent signaling pathways, is transported into the nucleus to promote downstream *ADAMTS5* transcription	Zhao et al. (2011)
	Chondrocyte differentiation		Gong et al. (2019)
**Notch signaling**	Chondrocyte differentiation	RBPjκ and MAM, which are activated by NICD from NOTCH receptors, can upregulate the expression of *HES1* and following *ADAMTS5*	Sugita et al. (2015)
			Saito and Tanaka, (2017)
**Wnt signaling**	Chondrocyte differentiation	BMP2-induced Wnt/β-catenin signaling promotes its downstream factor, LEF1, to bind to *ADAMTS5* promoter and to initiate its transcription	Papathanasiou et al. (2012)
**IL-1 signaling**	Inflammatory response	IL-1β can induce the overexpression of ADAMTS5 via AP-1/microRNA-30a (miR-30a) axis	Ji et al. (2016b)
**IL-6 signaling**	Inflammatory response	IL-6 can upregulate the expression of ADAMTS5 via the activation of downstream STAT3	Latourte et al. (2017)
**NF-κB signaling**	Inflammatory response	NF-κB, especially p65, stimulated by cytokines and ECM fragments can bind to the promoter of *ADMATS5* and upregulate its expression	Kobayashi et al. (2013)
	Mechanical stimulation	Gremlin-1 activated by excessive mechanical loading can activate NF-κB signaling, resulting in induction of ADAMTS5	Chang et al. (2019)
**SOX4 signaling**	Mechanical stimulation	SOX4 molecules induced by retinoic acid can directly bind to the promoter sequences of *ADAMTS5* and modulate its transcription	Takahata et al. (2019)
	Inflammatory response		

### Potential Therapies in OA Targeting ADAMTS5

In healthy articular cartilage, the balance between matrix synthesis and degradation is dynamically maintained. Overactivation of matrix remodeling and the inflammatory response are major events in synovial joints in the context of senescence, mechanical stress, and proinflammatory cytokines. In addition, analgesics and nonsteroidal anti‐inflammatory drugs are still clinical choices to relieve symptoms of OA (Alcaraz et al., 2019). No disease-modifying OA drugs have ever been applied in clinical treatment. Notably, in recent years, therapeutic options designed to modulate the expression and activity of ADAMTS5, for instance, monoclonal antibodies, small synthetic molecule inhibitors, small interfering RNAs (siRNAs), miRNAs, and injectable agents for ADAMTS5 blockade, have arisen as potential alternatives for OA treatment (Table 2).

**TABLE 2:** Potential drugs targeting ADAMTS5 in OA therapy.

Drug type	Drug name	Mechanism	Status	Reference
**Monoclonal antibodies**	CRB0017	CRB0017 binds to the spacer domain of ADAMTS5 and reduce its proteolytic activity	Preclinical	Chiusaroli et al. (2013)
	GSK2394002	GSK2394002 binds to catalytic/disintegrin-like domains	Preclinical	Larkin et al. (2015)
	2D3, 2D11, 2D5, and 2B9	2D3 and 2D11 react with epitopes in the catalytic/disintegrin-like domains of ADAMTS5	Discovery	Santamaria et al. (2015)
		2D5 binds to thrombospondin type 1 motif and 2B9 binds to the spacer domain		
	M6495	M6495 binds to the catalytic and/or disintegrin-like domain	Clinical (phase 1)	AS Siebuhr et al. (2020)
	Sheddase antibodies	Monoclonal antibodies selectively inhibit the LRP-1 sheddases to promote the endocytosis of ADAMTS5	Preclinical	Yamamoto et al. (2017)
	Syndecan 4 specific antibody	Injection of syndecan 4 specific antibody blockes ADAMTS5 protein maturation	Preclinical	Echtermeyer et al. (2009)
**Small molecule inhibitors**	AGG-523	A reversible, non-hydroxamate, zinc-binding selective inhibitor to both ADAMTS5 and ADAMTS4 developed by Wyeth/Pfizer	Discontinued (phase 1)	Chockalingam et al. (2011)
	Compound^a^	A series of compounds with carboxylate zinc-binding group	Discovery	Shiozaki et al. (2011)
	Compound 15f, 13g, 13e^b^	A series of nonclassical zinc-binding group compounds selected via encoded library technology	Discovery	Deng et al. (2012)
	Compound 7	A compound with zinc-binding group moieties in hydantoin series	Preclinical	Durham et al. (2017)
	GLPG1972	A compound with zinc-binding group moieties in hydantoin series	Clinical (phase 2)	Brebion et al. (2021)
	Glycoconjugated arylsulfonamide	A compound with positively charged residue-binding ability to the disintegrin-like domain of ADAMTS5	Discovery	Santamaria et al. (2021)
**RNAs**	ADAMTS5 siRNA	ADAMTS5 siRNA silences *ADAMTS5* gene by interfering with its mRNA translation	Preclinical	Chu et al. (2013)
				Hoshi et al. (2017)
	miRNA-140	miRNA-140 is located in one intron of *WWP2* gene and is a regulator of cartilage homeostasis	Preclinical	Si et al. (2017)
	WWP2 mRNA	WWP2 mRNA suppresses *ADAMTS5* upstream Runx2 signaling	Preclinical	Mokuda et al. (2019)
	ROR2 siRNA	ROR2 siRNA suppresses *ADAMTS5* upstream YAP/TAZ signaling	Preclinical	Thorup et al. (2020)
	Antisense oligonucleotides	Antisense oligonucleotides silences *ADAMTS5* gene by interfering with its mRNA translation	Preclinical	Garcia et al. (2019)

a (1S,2R, 3R)-2,3-Dimethyl-2-phenyl-1-sulfamidocyclopropanecarboxylates.

b The core structure of these compounds is triazine pyrrolidine (4-n-propanephenyl)sulfonamide.

### Monoclonal Antibodies and Small Molecule Inhibitors

Selective and high-affinity antibodies have been evaluated as direct attempts to block ADAMTS5 catalytic activity and reduce cartilage damage (Santamaria and de Groot, 2019). Antibody-based inhibitors, such as CRB0017, GSK2394002, and M6495, were selected and exhibited the efficacy of ADAMTS5 inhibition *in vivo* (Chiusaroli et al., 2013; Larkin et al., 2015; Santamaria et al., 2015; Siebuhr et al., 2020). M6495, which has completed phase 1 clinical trials, is an antibody that selectively binds to the catalytic metalloproteinase domain and inhibits ADAMTS5 *in vitro*, reducing aggrecan cleavage in OA joints (Siebuhr et al., 2020). However, due to potential side effects, most antibodies fail to progress beyond preclinical expectations, and only a few are undergoing or have progressed further than phase 1 clinical trials (Santamaria, 2020) (Table 2). For example, the risk of cardiovascular side effects was increased upon systemic administration of GSK2394002 in mice (Larkin et al., 2014). Since ADAMTS5 also exerts a role in cardiovascular and limb development (McCulloch et al., 2009; Dupuis et al., 2011), the long‐term impacts of these antibodies need to be investigated before clinical trials.

Apart from ADAMTS5-specific antibodies, other antibodies that block ADAMTS5 maturation and function also presented protective outcomes preclinically. Monoclonal antibodies that selectively inhibit LRP-1 (ADAMTS5 endocytic receptor) sheddases reversed OA cartilage degradation (Yamamoto et al., 2017). Intra-articular injection of inhibitors that block posttranslational modifications of the ADAMTS5 proprotein was also used to treat OA mice (Echtermeyer et al., 2009).

Compared with monoclonal antibodies, most small molecule inhibitors of ADAMTS5 are orally bioavailable (Shiozaki et al., 2011), while the specificity of small molecule inhibitors is not that exquisite. Small molecule inhibitors were selected based on the structure of the ADAMTS5 protein, and the majority of inhibitors were developed based on the catalytic metalloproteinase domain (Shiozaki et al., 2011; Chockalingam et al., 2011; Deng et al., 2012; Durham et al., 2017; Brebion et al., 2021; Nuti et al., 2013). The zinc-binding group in the catalytic metalloproteinase domain is the distinguishing structure of these inhibitors, such as hydroxamate and carboxylate (Shiozaki et al., 2011; Deng et al., 2012). However, zinc-binding domains widely exist in many metalloproteinases, which may lead to cross-inhibition of these drugs (Bakali et al., 2014). Many small molecule inhibitors were only tested at the discovery/preclinical stage or discontinued in phase 1 clinical trials (Table 2). GLPG1972 is a compound with zinc-binding group moieties and belongs to the hydantoin series (Brebion et al., 2021). After screening, *structure–activity relationship optimization* was used to improve its potency and eventually led to its discovery. GLPG1972 displayed high potency against ADAMTS5 in cultured cartilage explants and is now under phase 2 clinical trials with a high degree of selectivity (Brebion et al., 2021).

Since it is challenging to select suitable drugs among classical compounds with zinc-binding groups, new strategies are proposed to circumvent these drawbacks. Specific amino acid residues in the ancillary domains were targeted for drug development. For example, glycoconjugated arylsulfonamide was identified to target the disintegrin-like domain of ADAMTS5 with its positively charged residue-binding ability (Santamaria et al., 2021). This exosite inhibitor presented amenable selective inhibition of ADAMTS5 activity and indicated the prospects of a novel class of OA drugs.

### Posttranscriptional Suppression and Upstream Signaling Blockade of ADAMTS5 Using miRNAs and siRNA in OA Therapies.

Many antibodies and small molecule inhibitors failed to exhibit the expected results after preclinical testing. Thus, drugs with less cross-inhibition and off-target damage are required. miRNAs are a class of small, noncoding RNAs that specifically bind to messenger RNAs (mRNAs) and posttranscriptionally regulate protein expression level. miR-140 is an endogenous RNA abundantly expressed in chondrocytes and is located in one intron of the *WWP2* gene (Nakamura et al., 2008). Similar to the WWP2 protein, miRNA-140 helps maintain the homeostasis of cartilage, and miRNA-140 knockout mice showed OA-related changes (Miyaki et al., 2009; Miyaki et al., 2010). Multiple downstream factors, including ADAMTS5 and MMP13, were shown to be downregulated by miRNA-140 in OA model (Liang et al., 2016). Direct intra-articular injection of miRNA-140 has shown significant improvement of histological score of articular cartilage and significantly decreased expression levels of ADAMTS5 and MMP13 (Si et al., 2017). However, miRNA-140 can be degraded by nucleases under inflammatory conditions in OA. Chemical modifications, exosomes, viruses, and liposomes have been designed for the transport of miRNAs with the benefits of accurate delivery to targeted cells and slow release for cellular uptake (Duan et al., 2020). Tentatively, chitosan-mediated miRNA-140 and insulin-like growth factor 1 overexpression *in vivo* can significantly reduce ADAMTS5 and improve the repair of articular cartilage in OA (Zhao et al., 2019).

Similar to miRNAs, siRNAs are a class of double-stranded noncoding RNAs that bind to complementary mRNAs and promote their degradation. Lentivirus-mediated siRNA is used to knock down target genes (Tiscornia et al., 2003). The expression of ADAMTS5 was significantly decreased after injection of lentivirus-mediated ADAMTS5 siRNA *in vivo* and *in vitro* in a surgically induced OA mouse model (Chu et al., 2013). In addition, injection of ADAMTS5 siRNA without viral vectors also attenuated articular cartilage degeneration in an OA mouse model (Hoshi et al., 2017). Double-stranded siRNA needs to be unwound into a single-stranded component before binding to the target mRNA sequence (Chery, 2016). Antisense oligonucleotides, a class of single-stranded nucleic acids, are also introduced for posttranscriptional modification due to their higher affinity and selectivity, and lower toxicity after chemical modifications (Kole et al., 2012). Sustained local release of antisense oligonucleotides from a fibrin-hyaluronic acid hydrogel also resulted in long-term silencing of *ADAMTS5* in OA chondrocytes (Garcia et al., 2019).

In addition to directly knockdown ADAMTS5 translation, siRNAs that suppress ADAMTS5 upstream signaling were also used in OA treatment. Receptor tyrosine kinase–like orphan receptor 2 (ROR2) belongs to the tyrosine kinase receptor family and is involved in skeletal development (DeChiara et al., 2000). Thorup et al. (2020) demonstrated that blocking the activity of ROR2 can retard cartilage degradation in an OA mouse model by inhibiting YAP signaling (Thorup et al., 2020). ROR2 blockade also suppressed the expression of ADAMTS5 and protected mice from loss of cartilage integrity. In chondrocytes, ROR2 can facilitate YAP nuclear translocation and elevate BMP2 expression (Blaney Davidson et al., 2015). The results showed that decreased expression of ROR2 by intra-articular injection of ROR2 siRNA decreased downstream YAP signaling and ADAMTS5 expression. In addition to articular cartilage–protecting effect, ROR2 blockade achieved OA-induced pain relief and absence of side effects, at least until the mice were euthanized 22 weeks after birth (Thorup et al., 2020). Furthermore, WWP2 mRNA-treated chondrocytes also presented protective results via Runx2 signaling inhibition (Mokuda et al., 2019).

Compared with antibodies and small molecule inhibitors, miRNA- and siRNA-mediated ADAMTS5 inhibitory effects are specific due to complementary pairing. Considering that ADAMTS5 is involved in multiple regulatory mechanisms, nonspecific blockade of ADAMTS5 is not worth considering for OA therapy. Intra-articular injection of miRNA and siRNA can precisely knock down ADAMTS5 expression in imbalanced joints but also confines the drug to a limited space. Moreover, investigation on the specificity and bioavailability of RNA-based therapeutics in OA treatment are still challenging (Winkle et al., 2021). The off-target effects on tissues, cells, and genes may lead to severe toxicity or autoimmune responses (Hong et al., 2020). Besides, the instability and inefficient delivery of unmodified RNAs *in vivo* limit the improvement of therapeutic effect. To be noted, no clinical trials on OA have been registered so far (https://clinicaltrials.gov). However, recent years have seen a growing number of approvals for commercial use RNA therapies in treating liver, muscle, or the central nervous system diseases, shedding lights on further investigation of OA treatment (Crooke et al., 2019). The robustness of subcutaneous, intravitreal, and intrathecal delivery in hereditary transthyretin amyloidosis, cytomegalovirus retinitis, and spinal muscular atrophy treatment has provided perfect examples for a safe and efficient delivery of the therapeutic construct (Group, 2002; Aartsma-Rus, 2017; Wood, 2018; Gillmore et al., 2021). Progress in exploits of the molecular mechanisms of OA may facilitate the development and deployment of novel RNA therapeutics in future clinical trials.

## Summary and Outlook

In this review, we comprehensively discussed the roles of ADAMTS5 in OA development. ADAMTS5 is the main aggrecanase in the pathogenesis of OA and is the chief cause of articular cartilage breakdown and matrix loss. Under stimulation by inflammatory factors and mechanical stress overload, upstream signaling pathways function improperly, leading to dysregulation of ADAMTS5. A complex molecular signaling regulatory network modulates ADAMTS5-related OA pathogenesis. Since analgesics and nonsteroidal anti‐inflammatory drugs are still first-line options in OA therapy, disease-modifying OA drugs that inhibit ADAMTS5 expression and activity are required for OA therapies.
